# Patient Safety Culture Perception among Moroccan Healthcare professionals: Cross-Sectional Study in Public Hospitals

**DOI:** 10.4314/ejhs.v33i2.12

**Published:** 2023-03

**Authors:** Chaima Fihri Fassi, Yasmine Mourajid, Mohamed Chahboune, Abderraouf Hilali

**Affiliations:** 1 Hassan First University of Settat, Higher Institute of Health Sciences, Laboratory of Health Sciences and Technologies

**Keywords:** Patient safety, Perception, Patient safety culture

## Abstract

**Background:**

There is a growing recognition of the need to establish a culture that focuses on patient safety in order to reduce the number of adverse events associated with care and improve health-care quality in Morocco. The aim of this research is to analyze results of the perception of health professionals working in two university hospitals concerning the concept of patient safety culture in Morocco.

**Methods:**

This study evaluated the healthcare professional's perceptions of patient safety culture in two selected university hospitals centers in Morocco by using the validated French version of the Hospital Survey on Patient Safety Culture questionnaire (HSOPSC). A cross-sectional descriptive study was conducted in 2021. We randomly selected 10 health units of each hospital, to include up to 10 health professionals from each unit, regardless of length of experience. This self-administered questionnaire was distributed to a population of 204 Moroccan healthcare professionals who consisted predominately of available physicians and nurses across ten different health units.

**Result:**

The overall grade of patient safety was deemed “good” for 52 % of the staff, “very good” for 17%, against “failing” for 2%. Out of the 10 dimensions explored. The “Teamwork within units” dimension had the highest score with 80%. The dimensions with the lowest positive response rates were “Staffing (23%)”, “non-punitive response to error” (31%) and “Teamwork across units' (47%). Seven dimensions were considered underdeveloped and three were undeveloped.

**Conclusion:**

This work provides a better understanding of healthcare professional perception towards patient safety.

## Introduction

The culture of patient safety is an important component of healthcare quality and an issue whose notoriety has been of high concern globally for the world health organization (WHO) (1). As it is an elementary factor forming the behaviors, perceptions and attitudes of health professionals (2). It can alter the process of providing care and the effectiveness of protocols that lead to the successes of long-term treatments of the patients and prevention of medical error (3).

Adverse events are considered as damages emerging from errors or failures in the care-giving assistance provided by health professionals, whether intentionally or not, resulting in permanent or temporary harm injuries that incapacitate patients or even lead to death (4, 5).

Several studies demonstrate that adverse events and medical errors are influenced by patient safety culture (6–8). Hence, interventions for the reduction of adverse events incorporate an enhancement of patient safety culture and the establishment of a reporting system that permit to learn from errors without blame. Therefore, the perception of health professionals concerning PSC is considered primary to insure the prevention and evaluation of patient safety in the hospital units (9, 10).

In developing countries such as Morocco, the situation is more challenging, including higher risk of adverse events occurrence and underreporting, in consequence to the limitation in human resources, infrastructures, developed technologies, and lack of suitable measuring tools of the level and perception of safety in the health institutions (11–14). To our knowledge, no studies have assessed healthcare professional's perception of patient safety culture in Morocco, specifically in hospitals related to the University hospital center in Casablanca and the University hospital center in Rabat.

Therefore, considering the importance of strategies for the assumption of a positive patient safety culture, in these primary health institutions in Morocco, this research is aiming to assess the results of analysis of the perception of health professionals who work in university hospitals of the patient safety culture, conducted in response to the gap of knowledge on the subject in Morocco (15).

## Methods and Materials

**Study setting and participants**: This is a university hospital-based cross-sectional study in which the HSOPS French version questionnaire was used to assess patient safety culture from the healthcare professional perspective, conducted from January to March of 2021. The study was carried out in two university hospitals, one in Casablanca (Morocco) and another in Rabat (Morocco). Both institutions provide high-complexity care, teaching (undergraduate and graduate) and research functions in all medical specialties and has more than 3000 beds. We randomly selected 10 health units of each hospital, to include up to 10 health professionals from each unit, regardless of length of experience. The population (N =204) consisted of all staff (physicians, nurses, midwifes, and other health workers). The survey was administered to staff that interacted with the patients in the last 12 months. Participation in the study was voluntary.

**Study instrument**: The current research used as assessment tool the Hospital Survey on Patient Safety Culture (HSOPSC) questionnaire, which was created and developed by the Agency for Healthcare Research and Quality (AHRQ) (16, 17). The French version of the HSOPSC questionnaire explores the same constructs as the original version does, translated into French and validated by the Coordination Committee of the Clinical Evaluation and Quality in Aquitaine (CCCEQA) (18, 19). The questionnaire has shown acceptable psychometric properties in this version. It also has a good feasibility and acceptability as it was observed in several psychometric studies like Occelli and al (20, 21), reliability of the HSOPSC is reportedly high with a Cronbach alpha of 0.88 for the whole questionnaire and varies between 0.46 and 0.84 for the dimensions. Therefore, it was a valid and reliable tool to assess PSC.

The French version of the questionnaire is composed of Ten PSC dimensions explored through 45 items to assess the beliefs, skills and behaviors involved in the safety culture of the organization from hospital staff perspectives organized as follows:

D1: Overall perception of patient safety, D2: Frequency of events reported, D3: Supervisor/manager expectations and actions promoting patient safety, D4: Organizational Learning—Continuous Improvement, D5: Teamwork within units, D6: Communication openness, D7: Non-punitive response to error, D8: Staffing, D9: Management support for patient safety, D10: Teamwork across units, each dimension is composed of three to four items constructed in a positive or negative manner.

For each item, the respondent may choose a score on a five-point Likert scale with the response options ranging from (5) strongly agreeing to (1) strongly disagreeing. Scores (4) agree and (5) strongly agree are considered ‘positive’ in relation to PSC, score (3) is ‘neutral’ and scores (1) strongly disagree and (2) disagree are considered ‘negative’ in relation to PSC. The primary outcome was the percentage of positive responses for each dimension of the HSOPS. Negatively worded items were reverse-coded because an answer on a negatively worded item indicates a positive response.

Two other items assess individual assessments of patient safety: the “patient safety grade”, with response options of excellent, good, very good, poor and failing, and the “number of events reported”, with response options of no events reported, 1 to 2 events reported, 3 to 5 events reported, 6 to 10 events reported, 11 or more events reported.

**Data collection**: In this study, we distributed a paper-based questionnaire to the participants after obtaining their verbal consent. They could freely and anonymously fill in the questionnaire and return their responses directly to the investigator. According to the user guide of the French version of HSOPSC questionnaire, if none of the dimensions' sections were entirely filled, the questionnaire would not be taken into account. In addition, if less than half of the items in the questionnaire have been completed, or the same answers were given to all the items, the questionnaire would be illegible and excluded (20).

**Data analysis**: Data were entered and analyzed using the software Microsoft Excel. For general analysis of responses to different questions, we calculated the percentage of positive responses regarding the presence of patient safety culture in each dimension by dividing the number of positive responses (“strongly agree/agree”) by the total number of responses (positive, neutral and negative) in the dimensions.

A percentage of positive responses above 75% was considered developed, a percentage of positive responses between 75% and 50% was considered underdeveloped and a percentage below 50% was undeveloped, showing that there were issues that needed improvement. For items with reverse wording with a negative connotation, disagreement indicated a positive response. Thus, to calculate the percentage of positive responses among the answers, we needed to consider the strongly disagree/disagree responses as positive answer.

## Results

**Characteristics of the participants**: From the 255-targeted healthcare professionals that provided survey feedback professionals, 51 illegible questionnaires were excluded and in total 204 questionnaires were approved and analyzed. Therefore, the response rate was of 80%, which is a required criterion for significant results. The characteristics of health professionals including physicians, nurses, assistant, technicians and midwifes who responded to the questionnaire are summarized in (Table 1).

**Table 1:** Characteristics of participants

Characteristics	n	%
**Professional title**		
Physicians	56	27
Nurses	122	60
Assistant	3	1
Technicians and midwifes	23	11
**Seniority in the specialty or** **current occupation**		
Less than 1 year	35	17
1 to 2 years	44	22
3 to 5 years	49	24
6 to 10 years	40	20
11 years or more	36	18
**Seniority in the health institution**		
1 to 2 years	48	24
3 to 5 years	46	23
6 to 10 years	43	21
11 years or more	67	33
**Seniority in units**		
1 to 2 years	61	30
3 to 5 years	48	24
6 to 10 years	48	24
11 years or more	47	23
**Working time in the units**		
Less than 50% of working time	23	11
More than 50% of working time	181	89
**Participation in management** **committees**		
Yes	16	8
No	188	92

Healthcare professional's overall perception of patient safety quality ranked as good in 52% of cases (55% for physicians and 50% nurses) and poor in 23% of cases (20% for physicians and 29% for nurses). In addition, most professionals did not report any adverse events (62%) in the last 12 months (55% of physicians and 66% of nurses) as described in (Table 2) with the exception of professionals working for more than 10 years in the units or in a committee of management.

**Table 2:** Staff perception of patient safety quality and number of reported AEs during the last 12 months

Staff perception of patient safety quality	Overall %	Physicians %	Nurses %
Excellent	6	9	0
Very good	17	14	16
Good	52	55	50
Poor	23	20	29
Failing	2	2	5
**Number of events reported**	**Overall %**	**Physicians %**	**Nurses %**
No event reported	62	55	66
1 to 2 events reports	21	25	20
3 to 5 events reports	9	18	6
6 to 10 events reports	2	0	3
11 to 20 events reports	4	0	4
21 events reports or more	1	2	2

**PSC dimensions**: The overall perception of patient safety had an average positive score of 55%. Most dimensions had scores between 50% and 70% or above they are all underdeveloped or developed with the exception of three dimensions that were undeveloped with less than 50% scores (Table 3).

**Table 3:** The results of all PSC dimensions and items

Items of safety culture dimensions at the hospital’s units	Positive responses	
	
	Number	Percent
**D1: Overall perceptions of safety**	113	55
Patient safety is never sacrificed to get more work done	75	37
Our procedures and systems are good at preventing errors from happening	163	80
It is just by chance that more serious mistakes do not happen around here	50	25
We have patient safety problems in this facility	163	80
**D2: Frequency of events reported**	135	66
When a mistake is made, but is caught and corrected before affecting the patient, it is reported	119	58
When a mistake is made, but has no potential to harm the patient, it is reported	141	69
When a mistake is made that could harm the patient, but does not, it is reported	144	71
**D3: Supervisor/Manager expectations and actions promoting patient safety**	122	60
Manager says a good word when he/she sees a job done according to established patient safety procedures	147	72
Manager seriously considers staff suggestions for improving patient safety	151	74
Whenever pressure builds up, my manager wants us to work faster, even if it means taking shortcuts	77	38
My manager overlooks patient safety problems that happen over and over	113	55
**D4: Organizational learning and continuous improvement**	145	71
We are actively doing things to improve patient safety	161	79
Mistakes have led to positive changes here	143	70
After we make changes to improve patient safety, we evaluate their effectiveness	147	72
We are given feedback about changes put into place based on event reports	121	59
We are informed about errors that happen in the facility	149	73
In this facility, we discuss ways to prevent errors from happening again	146	72
**D5: Teamwork within units**	163	80
People support one another in this facility	160	78
When a lot of work needs to be done quickly, we work together as a team to get the work done	163	80
In facility, people treat each other with respect	165	81
When one area in this unit gets really busy, others help out	165	81
**D6: Communication openness**	136	67
Staff will freely speak up if they see something that may negatively affect patient care	166	81
Staff feel free to question the decisions or actions of those with more authority	132	65
Staff are afraid to ask questions when something does not seem right	111	54
**D7: Non-punitive response to error**	63	31
Staff feel like their mistakes are held against them	51	25
When an event is reported, it feels like the person is being written up, not the problem	74	36
We work in ‘crisis mode’ trying to do too much, too quickly	63	31
**D8: Staffing**	47	23
We have enough staff to handle the workload	65	32
Staff in this facility work longer hours than is best for patient care	34	17
We work in ‘crisis mode’ trying to do too much, too quickly	43	21
**D9: Management support for patient safety**	112	55
Management provides a work climate that promotes patient safety	105	51
The actions of management show that patient safety is a top priority	128	63
Management seems interested in patient safety only after an AE happens	66	32
Units work well together to provide the best care for patients	148	73
**D10: Teamwork across units**	95	47
There is good cooperation among units that need to work together	78	38
Units do not coordinate well with each other	77	38
It is often unpleasant to work with staff from other units	125	61
Things ‘fall between the cracks’ when transferring patients from one unit to another	112	55
Important patient care information is often lost during shift changes	116	57
Problems often occur in the exchange of information across units	62	30

The percentage of positive responses was highest for teamwork within units (80%) professionals felt that people supported each other, worked together as a team, and treated each other with respect. They had also the impression that in contact with their colleagues, they improved their care practice of safety. The lowest scores were (Table 3):

D8 (Staffing (23%)): the professionals felt there were insufficient staff members to handle the workload. Furthermore, they had the feeling of constantly working in urgency mode. D7 (Non-punitive response to error (31%)): staff focusing on the fear of attribution of responsibility of error to a single person. D10 (Teamwork across units (47%)): the staff underlined the existence of dysfunctions during inter-departmental exchanges and communication.

## Discussion

The present study was conducted to analyze healthcare professional's perception of PSC in two Moroccan university healthcare centers. Analyzing the perceptions of professionals working in health institutions units of care using a questionnaire makes it possible to approach the unit's safety culture, to discuss with the professionals the issues covered through the dimensions and make them aware of important items that need developing. Improving the safety of care is conditioned by a shared vision of professionals on a culture of safety (22, 23). The dimension of overall perception of safety had a score of (55%). This reflects the lack of safety standards and the implementation of strategic and corrective measures to increase awareness of this issue among health professionals in the two hospitals of this study.

Our study shows that one of the 10 dimensions explored, one was developed, six are considered underdeveloped and three undeveloped. The dimension of teamwork within units had the highest score, the staff communication within the units has proven to be of high quality in terms of coordination in care and supporting co-workers, freedom of expression is felt positively by the majority of staff interviewed. Most healthcare staff reported that they had shortage of staff to handle the workload, and that they worked longer hours than are recommended for patient care. This situation may have severe negative consequences for patient safety and quality of care (24). Furthermore, staff reported that they felt guilt about their mistakes, which were held against them, and the management focus was on their involvement in the AE rather than the AE itself. The issue of under-reporting AEs must become a priority to be taken into consideration and treated with vigilance; the staff should be encouraged to report AEs and rewarded for doing so (25,26).

Patient safety culture in university hospital centers has been given increasing attention and many studies have shown a low level of safety and high level of AEs with negative consequences in similar setting (27–30). In comparison with a study in 2020 of PSC in similar health institutions, the results of scores of the dimensions were similar to our study, the research of Fourar & al, in Algeria perceived that PSC was in overall underdeveloped. As well as comparable to our study non-developed dimensions were “non-punitive response to error” (31.9%), “Staffing” (26%), and “Teamwork across hospital units” with respective score of (39.5%). The dimension of teamwork within units had the highest score (78.5%) (31). In indication that the concept of patient safety culture and the perceptions and attitudes toward the improvement of this culture is a new concept that need more attention to create a safety environment in the health care institution in developing countries with similar sitting and sanitary challenges such as Algeria and Morocco.

According to healthcare organizations, for safety culture measurement to be useful, it must be accompanied by a return of the results at the same time in the units and at the institutional level (32).

We recommend four major areas for improvement that have been identified and will be subject to specific training:

1. Improving analysis and management of risk and medical error by training and raising staff awareness of the culture of safety and the report of AE, by requesting resources of the institution of health management team and by evaluating the progress of teams in units;

2. Developing the scientific knowledge of the staff, to propose actions for improvement and to mitigate the feeling of personalization of the error and blame culture by encouraging collective responsibility for the care and making a multifactorial and multidisciplinary analysis. The evaluation of improvement actions must be ensured.

3. Involvement of administrative staff in the problems of the units and an improvement communication between administrators and caregivers in order to adapt human resources in number and availability.

4. The continuous improvement of teamwork across units and quality of life at work for professionals, by implementing a better communication system across units.

Several benefits are expected in the long term, within the increase in reporting of AEs and the use of a more professional vocabulary, building the spontaneity of the team to integrate in a risk management approach, the increase feedback and analysis and improved communication between professionals across units. The effect of these measures will have to be reassessed by resubmitting this questionnaire to the teams in units.

Therefore, it is important to create a culture in which health professionals are encouraged and supported to identify and report AEs without fear of punitive action or blame. Reporting of AEs is an essential component of effective strategies to improve patient safety that includes identification of error, reporting, analysis and corrective actions (33).

This work provides a better understanding of healthcare professional perception towards patient safety. In general, the level of patient safety culture in hospitals is good. With 6 out of 10 dimensions underdeveloped according to the results of the questionnaire, the culture safety of care seems to be an axis of work and a priority in our university hospital centers. The next step will be to continually evaluate and implement actions of improvement targeting these issues like blame culture regarding adverse events reporting and lack of staff during shifts in the units. A pertinent perception of culture of security should make it possible to obtain the adherence of health professionals to the systems of safety of care. In fact, narrowing the communication gap across hospital department units, and providing an equal chance to everybody to give their input about the patient safety. The evaluation and development of a culture safety of care, it is a question of making safety a priority for everyone, professionals in the field as well as managers.

This study has several limitations, such as assessment of perception of PSC, using a self-administered questionnaire can be associated with a declaration bias. In addition, although we included two of the major university health centers of the targeted region, the chosen samples of professionals did not allow us to assume that these included settings were representative of the entire healthcare system in Morocco. Indeed, several respondents could not fill the questionnaire. In addition, we selected only one investigation tool to measure the patient safety culture, and hence the possibility of such biases cannot be completely dismissed.
